# Mir-421 and mir-550a-1 are potential prognostic markers in esophageal adenocarcinoma

**DOI:** 10.1186/s13062-022-00352-8

**Published:** 2023-02-24

**Authors:** Yun Ji, Lulu Wang, Guanglei Chang, Juan Yan, Liping Dai, Zhenyu Ji, Jingjing Liu, Meixia He, Hongliang Xu, Liguo Zhang

**Affiliations:** 1 BGI College, Zhengzhou University, No. 40 Daxue Road, Zhengzhou, 450007 China; 2 Henan Institute of Medical and Pharmaceutical Sciences, Zhengzhou University, No. 40 Daxue Road, Zhengzhou, 450052 China; 3 Department of Neurology, The First Affiliated Hospital of Zhengzhou University, Zhengzhou University, Zhengzhou, 450000 China; 4 Laboratory of Tumor Molecular Biomarkers, Zhengzhou University, Zhengzhou, 450000 China; 5 Center For Disease Control And Prevention, Health Bureau of Menglian Daizu Lahuzu Wazu Autonomous County, Pu’er Menglian, 665800, China

**Keywords:** miRNA, Mir-421, Mir-550a-1, Prognostic marker, Esophageal adenocarcinoma

## Abstract

**Objective:**

To identify the prognostic indicators of esophageal adenocarcinoma (EAC) for future EAC diagnosis and treatment.

**Methods:**

The EAC dataset from The Cancer Genome Atlas was screened for differentially expressed microRNAs (miRNAs) and mRNAs associated with EAC. Weighted gene coexpression network analysis was performed to cluster miRNAs or mRNA with similar expression patterns to identify the miRNAs or mRNA that are highly associated with EAC. Prognostic miRNAs for overall survival (OS) were identified using Cox proportional-hazards regression analysis and least absolute shrinkage and selection operator based on survival duration and status. Two types of miRNAs were selected to develop a prognostic signature model for EAC using multiple Cox regression analysis. Furthermore, the signature was validated using internal validation sets 1 and 2. The receiver operating characteristic curve and concordance index were used to evaluate the accuracy of the signature and validation sets. The expression of miR-421, miR-550a-3p, and miR-550a-5p was assessed using quantitative polymerase chain reaction (qPCR). The proliferation, invasion, and migration of EAC cells were assessed using CCK8 and transwell assays. The OS of target mRNAs was assessed using Kaplan–Meier analysis. Functional enrichment analysis of the target mRNAs was performed using Metascape.

**Results:**

The prognostic signature and validation sets comprising mir-421 and mir-550a-1 had favorable predictive power in OS. Compared with the patients with EAC in the high-expression group, those assigned to the low-expression group displayed increased OS according to survival analysis. Differential and qPCR analysis showed that miR-421, miR-550a-3p, and miR-550a-5p were highly expressed in the EAC tissues and cell lines. Moreover, the downregulation of miR-421 and miR-550a-3p with inhibitor markedly suppressed the proliferation, invasion, and migration in OE33 cells compared with the negative control. A total of 20 target mRNAs of three miRNAs were predicted, among which seven target mRNAs—*ASAP3*, *BCL2L2*, *LMF1*, *PPM1L*, *PTPN21*, *SLC18A2,* and *NR3C2*—had prognostic value; *PRKACB*, *PDCD4*, *RPS6KA5*, and *BCL2L2* were enriched in the miRNA cancer pathway.

**Conclusion:**

Prognostic indicators of EAC may be useful in future EAC diagnosis and treatment.

**Supplementary Information:**

The online version contains supplementary material available at 10.1186/s13062-022-00352-8..

## Introduction

The Global Cancer Statistics 2018 report revealed that esophageal carcinoma (ESCA), which is one of the most common malignant cancers worldwide, ranked seventh in terms of cancer incidence and sixth in terms of cancer-associated mortality globally [1]. ESCA can be divided into esophageal adenocarcinoma (EAC) and esophageal squamous carcinoma [2]. The incidence of EAC has increased substantially over the recent decades, and it remains a highly fatal disease with a poor prognosis [3, 4]. The prognostic outcomes for EAC is unfavorable, and the 5-year overall survival (OS) is < 15% despite the advancements in diagnosis and treatment [5, 6]. Exploring the mechanism that leads to the development and progression of EAC would be significant in understanding the poor outcomes of EAC. Thus, there is an urgent need to identify more novel and potential indicators that can help understand EAC and improve decision-making in clinical practice to effectively predict the occurrence, progression, and prognosis of this disease [7].

MicroRNAs (miRNAs) are noncoding RNAs that play an important role in cell differentiation, proliferation, and survival by binding to complementary target mRNAs, leading to the inhibition or degradation of mRNA translation [8]. Increasing evidence has indicated that miRNAs can be used in the diagnosis and prognosis of various tumors and diseases, including colorectal cancer, cutaneous melanoma, acute coronary syndrome, and acute myeloid leukemia; however, these are not part of standard clinical practice [9–12].

Studies reporting the miRNA biomarkers that improve the diagnosis and treatment of EAC are limited. Therefore, identification of new diagnostic and prognostic biomarkers is urgently needed because of the biological complexity and unfavorable prognosis of EAC.

In the past decade, novel biomarkers and therapeutic targets in various cancers have been explored using microarray and next-generation sequencing technologies [13–15]. However, the analyses of related makers are inadequate and even contradictory because of the different statistical data processing methods used [16]. To solve this problem, integrated bioinformatics methods such as weighted gene coexpression network analysis (WGCNA) have been used in various cancer studies; these were initially used to identify miRNA modules that are highly associated with EAC [17–19].

**Fig. 1:**
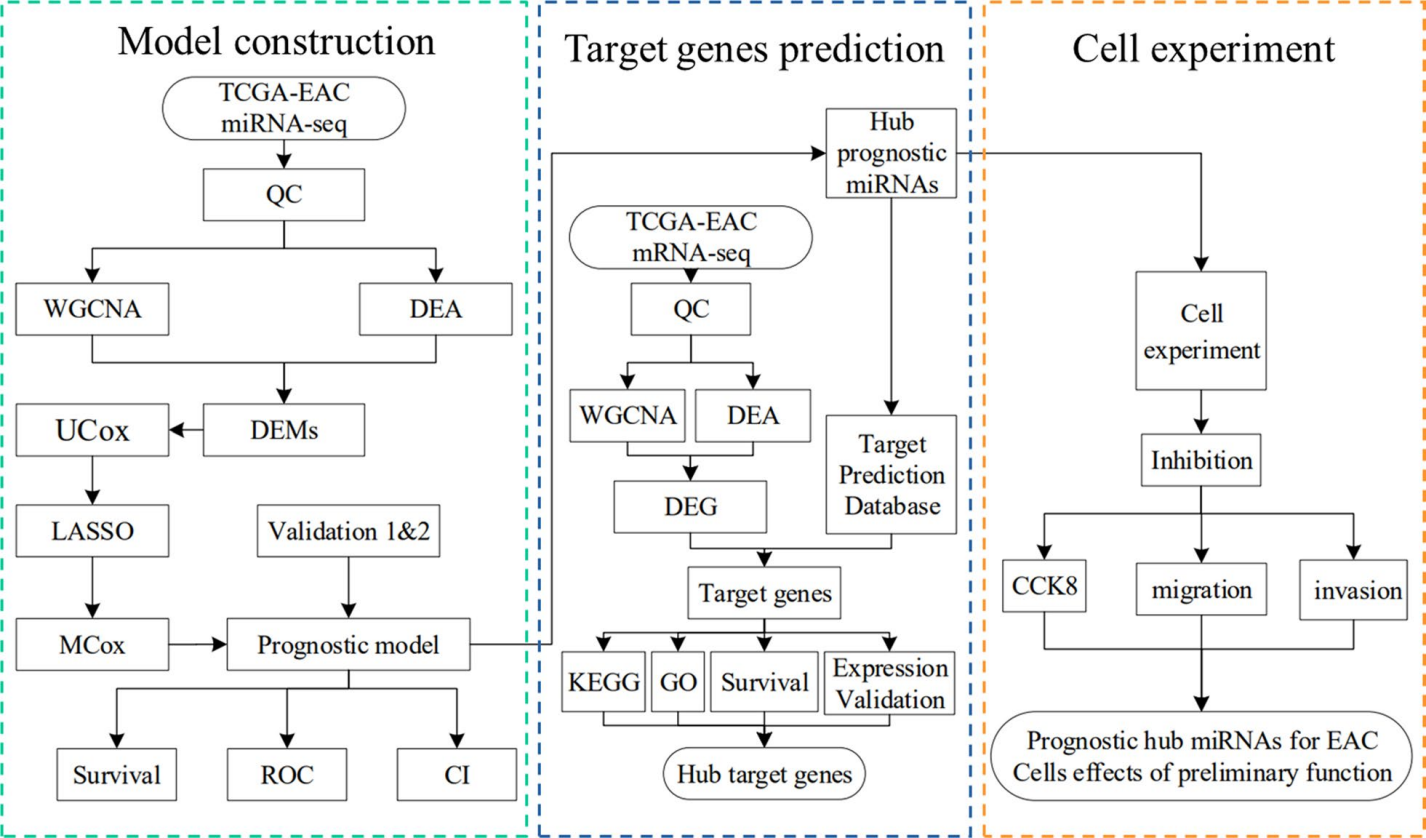
The entire analysis flow chart. *QC* Quality control, *DEA* Differentially expressed analysis, *DEMs* Differentially expressed miRNAs, *UCox* Univariate Cox,* MCox* Multivariate Cox

In this study, a prognostic model of a signature comprising two miRNAs was constructed based on a miRNA dataset from The Cancer Genome Atlas (TCGA); two prognostic miRNAs were screened for the prognosis of EAC. Moreover, the influence of two prognostic miRNAs on the proliferation, invasion, and migration of EAC cells was assessed. The Metascape database was explored for the possible cellular functions and pathways of target mRNAs related to this signature. Furthermore, seven prognostic target mRNAs were identified through Kaplan–Meier analysis. The entire analysis flow chart is shown in Fig. 1.

## Materials and methods

### Data analysis using the EAC cohort of TCGA

The miRNA-Seq data of 88 tumor tissues and 9 adjacent nontumorous tissues (Additional file 1: 1) and clinical information for EAC (Additional file 1: 2) were derived from TCGA (https://tcga-data.nci.nih.gov/tcga/) at the miRNA gene level rather than at mature miRNA level on October 19, 2020. For clinical data, clinical information from normal patients was deleted. Patient miRNAs with a mean value of expression = 0 were removed.

### Screening of differentially expressed miRNAs and genes

To identify differentially expressed miRNAs (DEMs) and differentially expressed genes (DEGs) in EAC and normal samples, edgeR was performed to normalize the gene read counts to the trimmed mean of M values [20]. *P*–values were adjusted to minimize the false discovery rate (FDR) using the Benjamini & Hochberg method [21]. A |Fold change| of > 1 and adjusted *P*-value < 0.05 were considered significant [22].

To identify DEMs and DEGs associated with EAC, WGCNA [23]—an algorithm used to identify gene coexpression network via high-throughput expression profiles with different traits—was performed to cluster miRNAs with similar expression patterns for obtaining the most related miRNAs from patients with EAC. WGCNA with a softpower of 2 and module size cutoff of 15 were set as the threshold for miRNAs, whereas a softpower of 10 and module size cutoff of 100 were set as the threshold on the basis of the expression values of all mRNAs.

The VennDiagram package [24] was used to intersect the results of WGCNA and DEMs identified to screen for DEMs associated with EAC.

### Construction of a multimicroRNA-based classifier

Univariate Cox proportional-hazards regression was used to identify the relationship between the DEM expression and OS of 88 patients. To prevent data overfitting, miRNAs with a *P*-value of < 0.05 (candidate variables) were rescreened via Lasso Cox regression analysis and the sample with a survival time of = 0 was removed [25]. Thereafter, stepwise multivariate Cox proportional-hazards regression analysis was performed to evaluate the contribution of the selected miRNAs. The regression methods were used to construct a classifier. Using a multivariate Cox regression model to calculate the risk scores for all patients and considering the median risk score as the cutoff value, the patients were divided into high- and low-risk groups. The analysis was performed using the survival R package (version 2.38, https://CRAN.R-project.org/package=survival) and rms (version 6.1.1, https://CRAN.R-project.org/package=rms). The R package “survivalROC” (version 1.0.3, https://CRAN.R-project.org/package=survivalROC), which performs receiver operating characteristic curve (ROC) and concordance index (CI) analyses using 1-, 2-, 3-, 4-, 5- OS data, was used to assess the performance of the prognostic signature model. The R package “caret” was used to randomly assign 43 of the patients to the internal training set and 44 to the internal testing set. Furthermore, the model of prognostic signature was validated using internal validation sets 1 and 2.

### Kaplan–Meier survival analysis

The Kaplan–Meier plotter, a database that integrates the resources of TCGA and Gene Expression Omnibus, evaluates the effects of mRNAs, miRNAs, and proteins on patient survival in 21 cancer types (http://kmplot.com/). The Kaplan–Meier plotter database was used to analyze the effects of miRNAs and their target genes on patient survival in EAC.

### Expressions in the GC subgroup and target gene prediction for microRNAs

The miRbase was used to obtain mature miRNAs of precursor miRNAs (premiRNA). The miRDB (http://www.mirdb.org/), R and Hiplot (https://hiplot.com.cn) were performed to reshape and visual data of expressions in the GC subgroup for mature miRNAs, respectively. The miRWalk (http://mirwalk.umm.uni-heidelberg.de/), miRTarBase (http://mirtarbase.mbc.nctu.edu.tw/), and TargetScan (http://www.targetscan.org/) databases were used to explore target mRNAs. The Cytoscape software (Version 3.7.2; http://www.cytoscape.org/) was used to visualize and construct the miRNA–mRNA network.

### Functional enrichment analysis

To determine the biological functions and potential signaling pathways of overlapping target mRNAs, gene ontology (GO) and Kyoto Encyclopedia of Genes and Genomes (KEGG) pathway enrichment analyses were performed using the Metascape database (http://metascape.org/gp/index.html#/main/step1).

### Cell culture and real-time quantitative polymerase chain reaction

A normal human esophageal epithelium cell line (Heec) purchased from the Chinese National Infrastructure of Cell Line Resource, and two EAC cell lines (OE33 and SEG-1) were purchased from the Chinese Binsui Bio for in vitro validation.

OE33 cells were cultured in complete medium comprising 90% Dulbecco's modified Eagle’s medium (Invitrogen, Carlsbad, USA) and 10% fetal bovine serum (HyClone, USA). SEG-1 and Heec cells were cultured in Roswell Park Memorial Institute Medium-1640 (Invitrogen, Carlsbad, CA, USA).

TRIzol (Solarbio, China) was used to isolate total RNA from whole-cell lysates using the PrimeScript™ RT Reagent Kit (TaKaRa, USA). Bulge-LoopTM miRNA qRT-PCR primers were used to synthesize cDNA according to the manufacturer’s instructions. Real-time quantitative polymerase chain reaction (qRT-PCR) was performed using Takara Bio Green Premix Ex Taq™ II (Tli RNaseH Plus, TaKaRa, USA) following the manufacturer’s instructions. U6 was used as the endogenous control. All primers were obtained from RiboBio Co., Ltd., China.

### miRNA transfection

miR-421 and miR-550a-3p inhibitors and corresponding controls were synthesized and purchased from RiboBio. Cells were cultured overnight in six-well plates (2 × 10^5^ cells/well) and then transfected with miR-421 inhibitor (concentration, 100 nM), miR-550a-3p (100 nM), and corresponding negative control (100 nM) when the cell confluence reached 30–40%. The transfection reagent LipofectamineTM3000 (Invitrogen, USA) was used.

### CCK8 assay

Approximately 2 × 10^3^ cells per chamber were cultured in 24-well plates, and then the CCK8 assay was performed using the CCK8 kit (Solarbio, China). A microplate reader (Bio-Rad, USA) was used to measure the absorbance at an optical density of 450 nm.

### Transwell invasion and migration assay

The mobility of transfected cells was detected using transwell chambers (Corning, China); for migration, 5 × 10^4^ cells per chamber were cultured in transwell chambers without basement membrane coating. For invasion, 1 × 10^5^ cells/chamber were cultured in transwell chambers containing a basement membrane coating. Afterward, the cells were incubated for 24 h in an incubator at 37 °C with 5% CO_2_; the cells of the inner chamber were wiped using a swab and cells on the outer chamber were fixed using methanol for 15 min and stained using 0.5% crystal violet (Solarbio, China) for 30 min. Then, the cells were washed three or four times with phosphate buffered saline and observed under an inverted phase microscope (ZEISS, Germany) to obtain field images.

### Statistical analysis

The pairwise Pearson correlation was used to assess the weighted coexpression relationship among all dataset subjects in an adjacency matrix. Logrank *P*-value was used to analyze the differences between the low- and high-risk/expression groups in survival analyses. Differences between the variables were considered significant if |log2FC|≥ 1 and adjusted *P*-value < 0.05 in difference expression analysis or A fold change of > 1 and *P*-value < 0.05 in qRT-PCR analysis. The *R* software (version 4.0.1) was used.

## Results

### Identification of DEMs associated with EAC

**Fig. 2:**
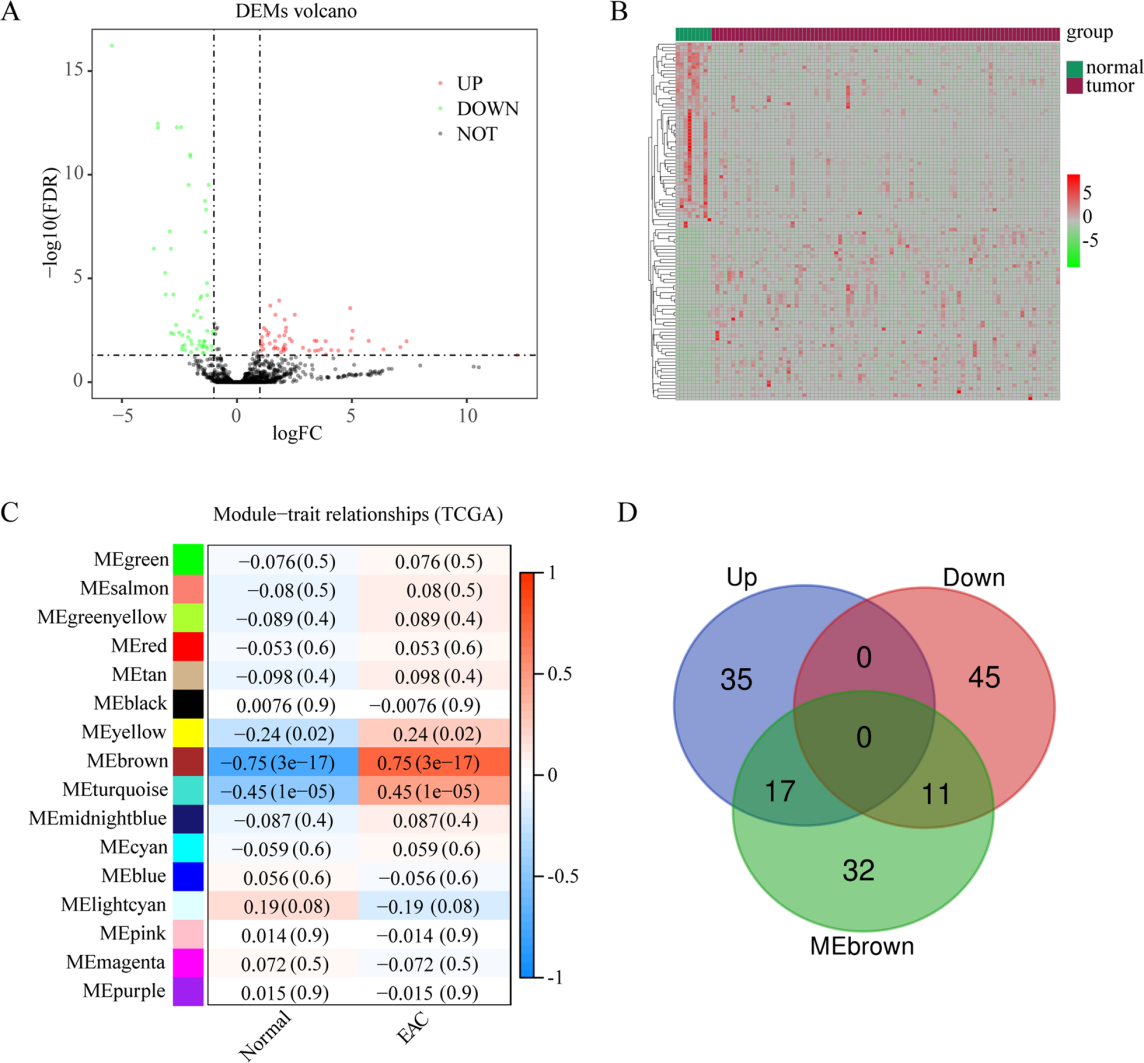
Identification of differentially expressed microRNAs (DEMs) associated with esophageal adenocarcinoma (EAC). **A** Volcano plots of DEMs. **B** Heatmap of DEMs in both EAC and adjacent normal samples. **C** Relationship of DEMs between the modules of the normal samples and those of EAC samples. **D** Venn plots of microRNAs between DEMs and microRNAs in the brown module

DEMs obtained from TCGA included 52 upregulated and 56 downregulated miRNAs (Fig. 2A–B). Using WGCNA, 16 coexpression modules were identified, among which a brown module, which included 60 miRNAs, showed a high association with EAC (Fig. 2C). To obtain the DEMs associated with EAC, 28 miRNAs were obtained using data between DEMs and the brown module, including 17 upregulated and 11 downregulated miRNAs identified using the VennDiagram package in R (Fig. 2D).

### Identification of prognostic microRNA signatures

**Fig. 3:**
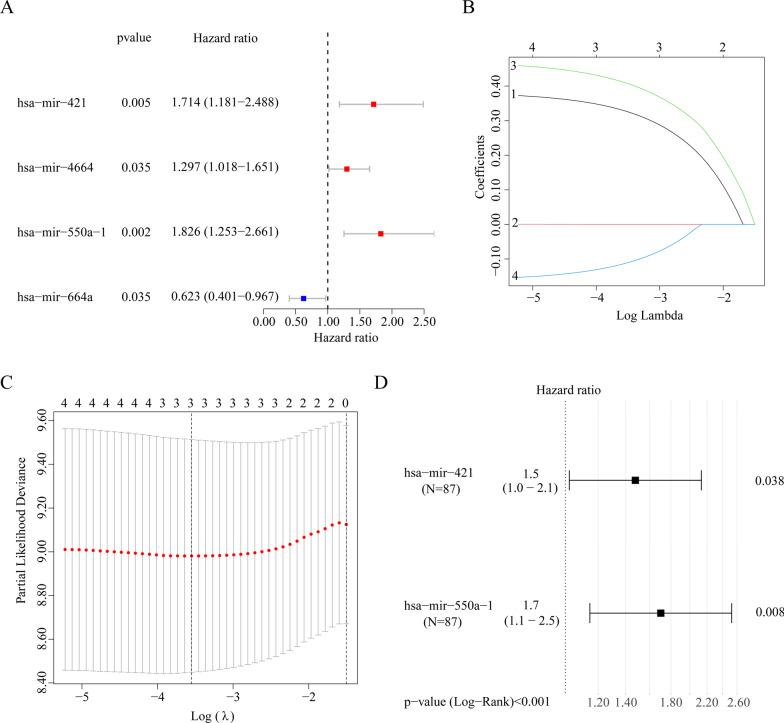
Identification of prognostic microRNAs (miRNAs). **A** Univariate Cox regression analysis of four differentially expressed miRNAs (DEMs). **B**, **C** Lasso-penalized Cox regression analysis of four DEMs. **D** Multivariate Cox regression analysis of three DEMs

A total of 4 out of 28 significant miRNAs associated with the expression levels in patients with EAC were found to be significantly related to the OS on univariate Cox regression analysis (Fig. 3A). Following initial filtration, Lasso penal Cox analysis with penalty parameters adjusted by tenfold cross-validation was performed to further narrow the mRNAs by selecting those that occurred > 900 times out of a total of 1000 replicates (Fig. 3B–C). Thus, three miRNAs were identified. Multivariate Cox proportional-hazards regression model analysis revealed that the prognostic signature contained the following two genes: has-mir-550a-1 and has-mir-421 (Table 1).

**Table 1:** Prognostic value of the two microRNAs in patients with esophageal adenocarcinoma in The Cancer Genome Atlas cohort

Symbol	Log_2_FC	Univariate analysis		Multivariate analysis		
		HR (95% CI)	*P*-value	HR (95% CI)	*P*-value	Coefficient
mir-421	1.42	1.714(1.202–2.558)	0.005	1.490(1.029–2.158)	0.038	0.399
mir-550a-1	1.84	1.826(1.329–2.900)	0.002	1.734(1.219–2.759)	0.004	0.606

*HR* Hazard ratio, *CI* Confidence interval

**Fig. 4:**
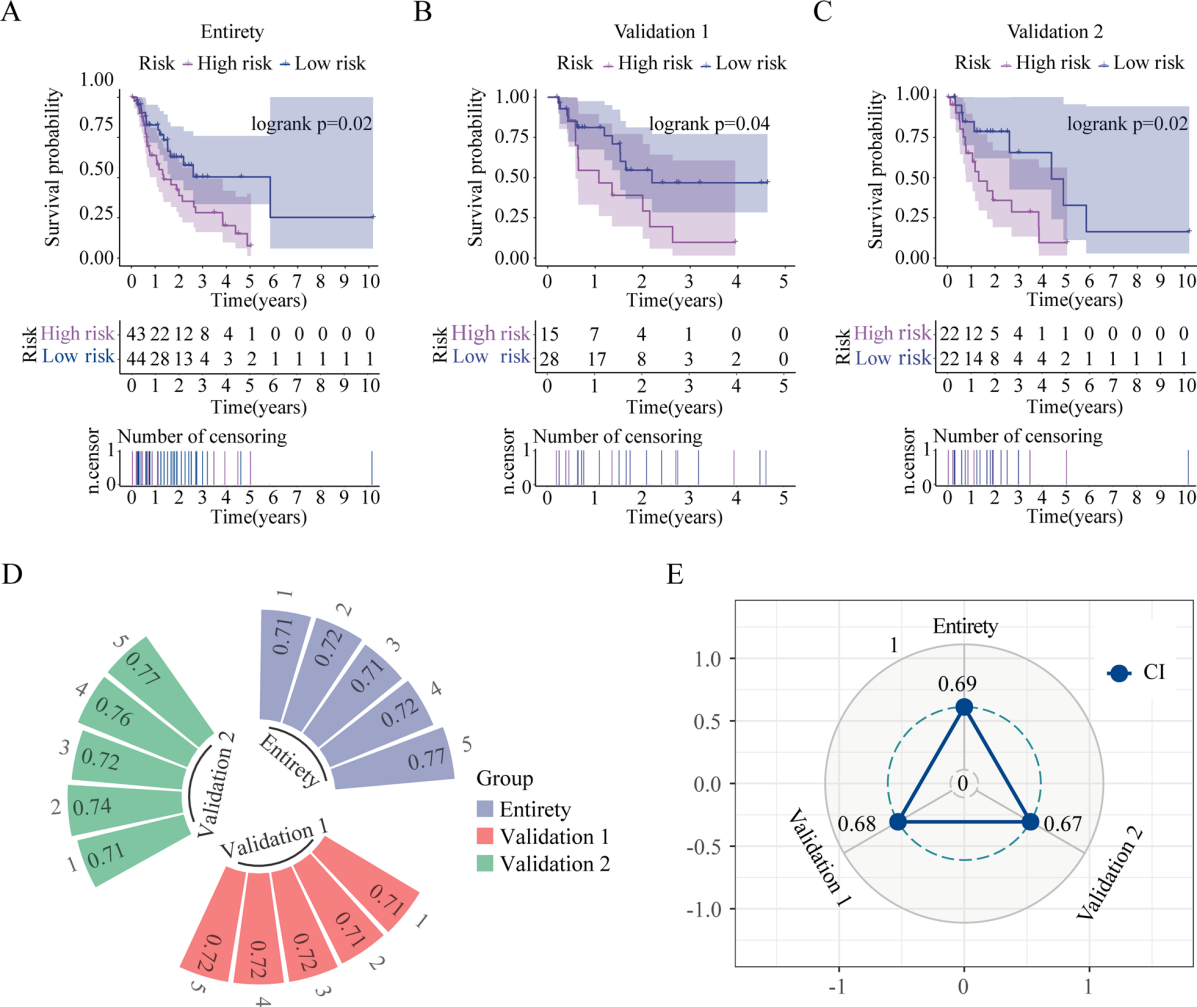
Kaplan–Meier curves and prediction of signature model and validation sets for the low- and high-risk groups

Interestingly, all miRNAs had a hazard ratio of > 1 and were considered prognostic risk factors (Fig. 3D). The results of prognostic risk score, survival status, and expression of the two OS-related miRNAs for patients with EAC were displayed in (Additional file 3: Fig. 1A–C). Kaplan–Meier survival analysis indicated that the OS of patients in the low-risk groups was remarkably high compared with that of those in the high-risk groups, implying that the high-risk groups were associated with a poor prognosis (Fig. 4A–C). To evaluate the prognostic model, the 1-, 2-, 3-, 4- and 5-year ROC curves and CI values of entirety as well as internal validation sets 1 and 2 were plotted. The area under the curve (AUC) values of 1-, 2-, 3-, 4-, and 5-year entirety were 0.71, 0.72, 0.71, 0.72, and 0.77, respectively (Fig. 4D). The AUC of internal validation sets 1 and 2 were > 0.7. Furthermore, the CI values of entirety as well as validation sets 1 and 2 were 0.69, 0.68, and 0.67, respectively. (Fig. 4E). These results implied that the prognostic gene signature performed well in survival prediction.

### OS of prognostic miRNAs

**Fig. 5:**
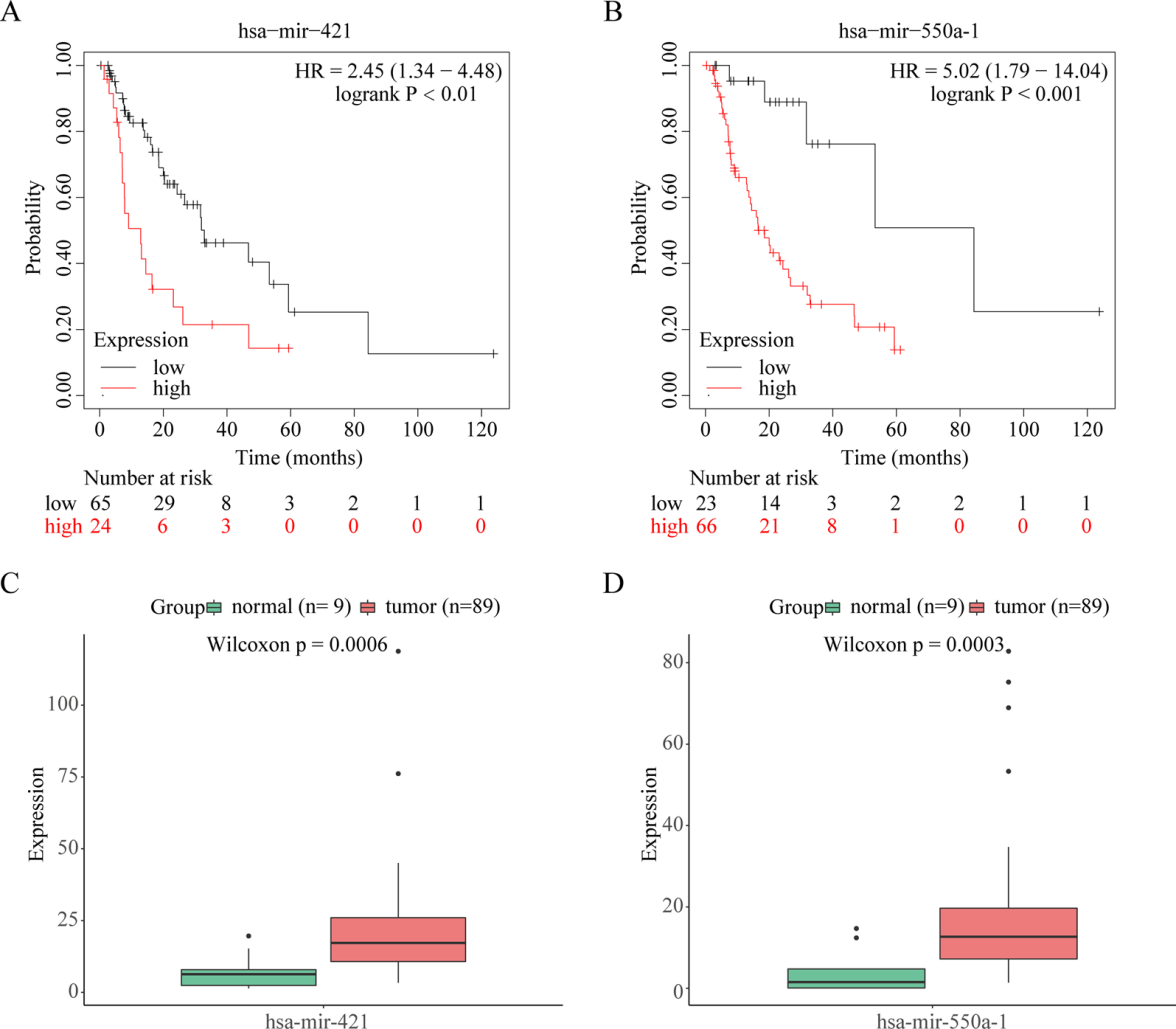
Overall survival (OS) and expression analyses. **A**, **B** OS of mir-421 and mir-550a-1, respectively. **C**, **D** Expression value of mir-421 and mir-550a-1, respectively

Kaplan–Meier analysis based on optimal cut-off value of miRNAs expression was used to explore the prognostic information of mir-421 and mir-550a-1 in order to identify OS. The OS of two miRNAs was poorer in the high-expression group than in the low-expression group (*P* < 0.05; Fig. 5A–B). To further ensure the accuracy of the results, the median was designed to divide expression grouping of EAC patients. The results showed that patients with high expression had a lower overall survival than those with low expression (Additional file 3: Fig. 2). Previous results have shown that mir-421 and mir-550a-1 show a higher expression in EAC samples than in paracancerous samples (Fig. 5C–D). Therefore, these data indicate that mir-421 and mir-550a-1 have prognostic value for patients with EAC.

### Verification of the expression of miR-421, miR-550a-3p, and miR-550a-5p

**Fig. 6:**
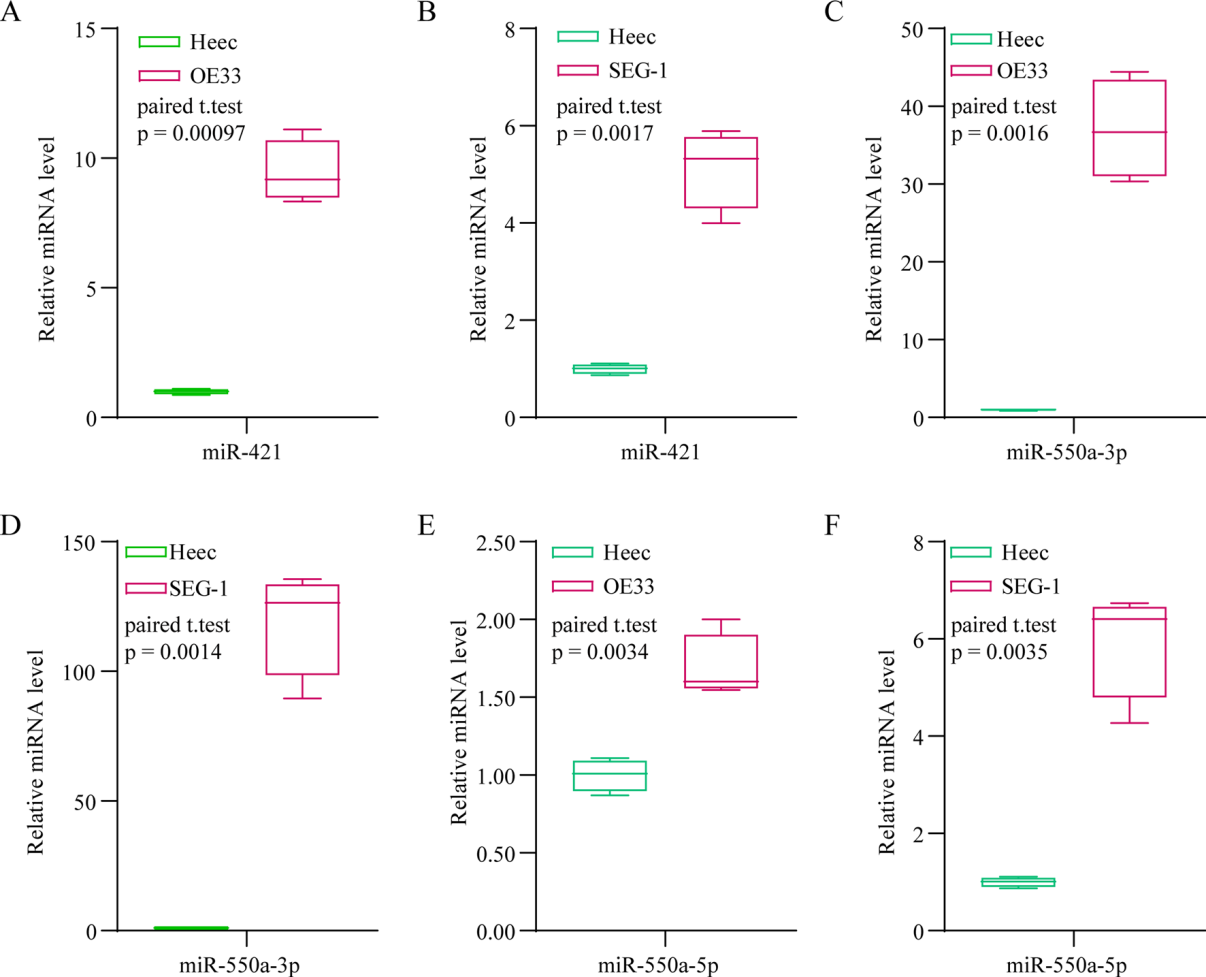
Expression of prognostic microRNAs indicators in the cell lines

The mature miRNAs of premiRNAs, miR-421, miR-550a-3p, and miR-550a-5p were identified using miRbase database, and the expression of Mature mir-421 and mir-550a-1 in the EAC subgroup was analyzed. As a result, they expressions are correlated with the vast majority of tumor grade, stage, M, N, T and gender compared with normal samples (Additional file 3: Fig. 3 and Additional file 3: Fig. 4). Importantly, the expression of miR-421, miR-550a-3p, and miR-550a-5p was verified using qRT-PCR analysis of Heec, OE33, and SEG-1 cells. The expression of these miRNAs was higher in OE33 and SEG-1 cells than in Heec cells (Fig. 6). Because the miR-550a-3p expression was the highest in miR550a-3p and miR-550a-5p, miR-550a-3p and miR-421 were selected for the subsequent experiments.

### Suppression of miR-421 and miR550a-3p inhibited the proliferation, invasion, and migration of EAC cells

**Fig. 7:**
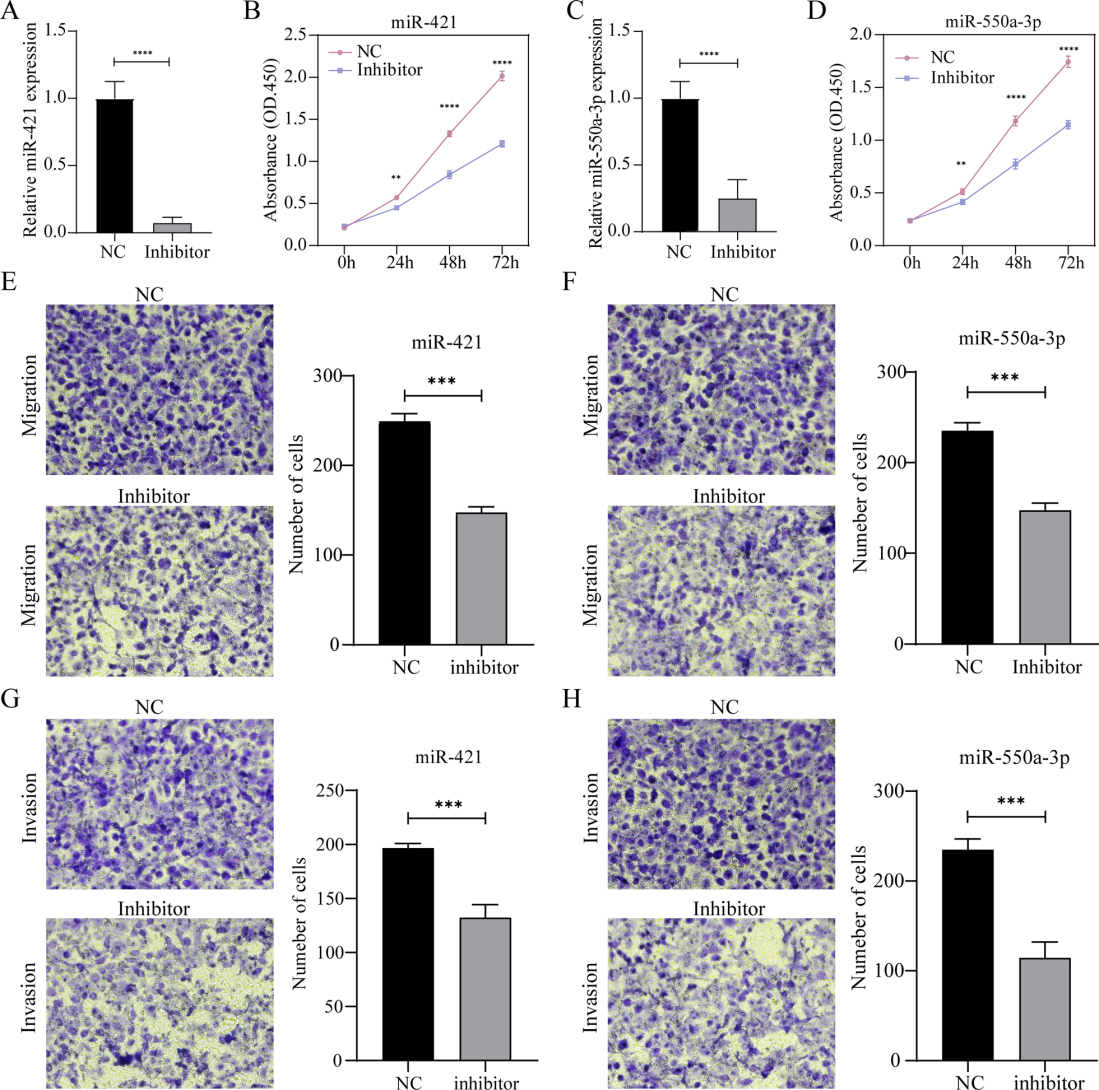
Effects of the suppression of miR-421 and miR-550a-3p on the proliferation, invasion, and migration of OE33 cells. Paired *t* test *P < 0.05, **P < 0.01, and ***P < 0.001

miR-421 and miR-550a-3p inhibitors were transfected into OE33 cells. The results showed that the downregulation of miR-421 and miR-550a-3p with inhibitor markedly suppressed the proliferation (Fig. 7A–D), migration (Figs. 7E and F), and invasion (Figs. 7G and H) of OE33 cells compared with the negative control.

### Identification of DEGs associated with EAC

Differential analysis showed that 3,390 mRNAs were DEGs according to the critical value (FDR < 0.05 and |log2FC|> 1). Of these, 1,155 were upregulated and 2,235 were downregulated (Additional file 3: Fig. 5A). Moreover, the distribution of the significant DEGs was assessed via heatmapping (Additional file 3: Fig. 5B).

Five mRNA modules were identified using WGCNA (Additional file 3: Fig. 5C). Among these five mRNA modules, the blue module had a strongly-connected gene coexpression network with EAC (Additional file 3: Fig. 5C). A total of 610 DEGs associated with EAC were obtained from the genes between DEGs and the blue module, including 432 upregulated and 178 downregulated mRNAs (Additional file 3: Fig. 5D).

### Construction of the miRNA–mRNA network and functional enrichment analysis of downregulated target mRNAs

**Fig. 8:**
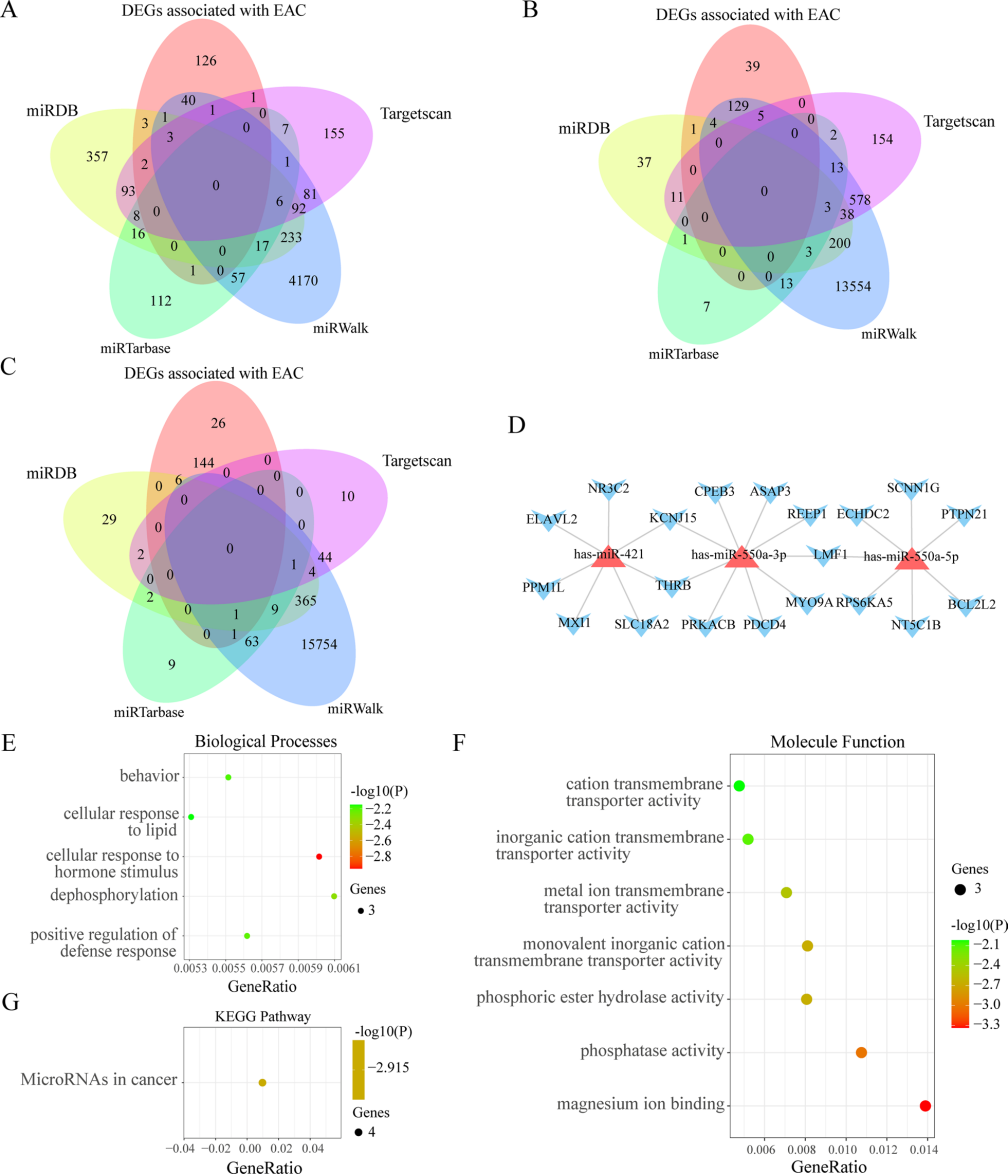
Prediction of target mRNAs and establishment of a microRNA (miRNA)–mRNA axis and Gene ontology enrichment and Kyoto Encyclopedia of Genes and Genomes (KEGG) pathway analysis of the 20 target mRNAs. **A**, **B**, and **C** overlapped target mRNAs were analyzed using the predicted target mRNAs, differentially expressed genes (DEGs) associated with esophageal adenocarcinoma, and significantly-downregulated mRNAs, respectively. **D** miRNA–mRNA network was constructed using the three miRNAs for EAC prognosis and 20 overlapped target mRNAs. **E** Biological process. **F** Molecular function. **G** KEGG pathways

Upregulated miRNAs are known to promote or inhibit the occurrence and progression of tumors by downregulated target mRNAs. Therefore, we used these downregulated target genes for subsequent analysis. The downstream target mRNAs with reference to the three mature miRNAs are identified using miRDB, miRTarBase, miRWalk, and TargetScan. Moreover, potential mRNAs, which were only shared by the downregulated DEGs associated with EAC and at least two databases, were selected to enhance the accuracy of the prediction (Fig. 8A–C). The results revealed an association between the miRNAs and their corresponding 20 target mRNAs (Fig. 8D).

GO enrichment analysis of biological processes indicated that the target mRNAs of three maturing miRNAs were mainly enriched in behavior, cellular response to lipid, cellular response to hormone stimulus, dephosphorylation, and positive regulation of defense response (Fig. 8E). Figure 8E shows the enriched molecular function GO terms. Unfortunately, cellular components were not enriched when the *P*-value was < 0.01. The KEGG pathway analysis revealed that the target mRNAs participate in miRNAs in cancer pathways (Fig. 8G). Therefore, these target mRNAs may be associated with cellular response, transmembrane transporter activities, and miRNAs in cancer pathways.

### Survival analysis of downregulated target mRNAs

**Fig. 9:**
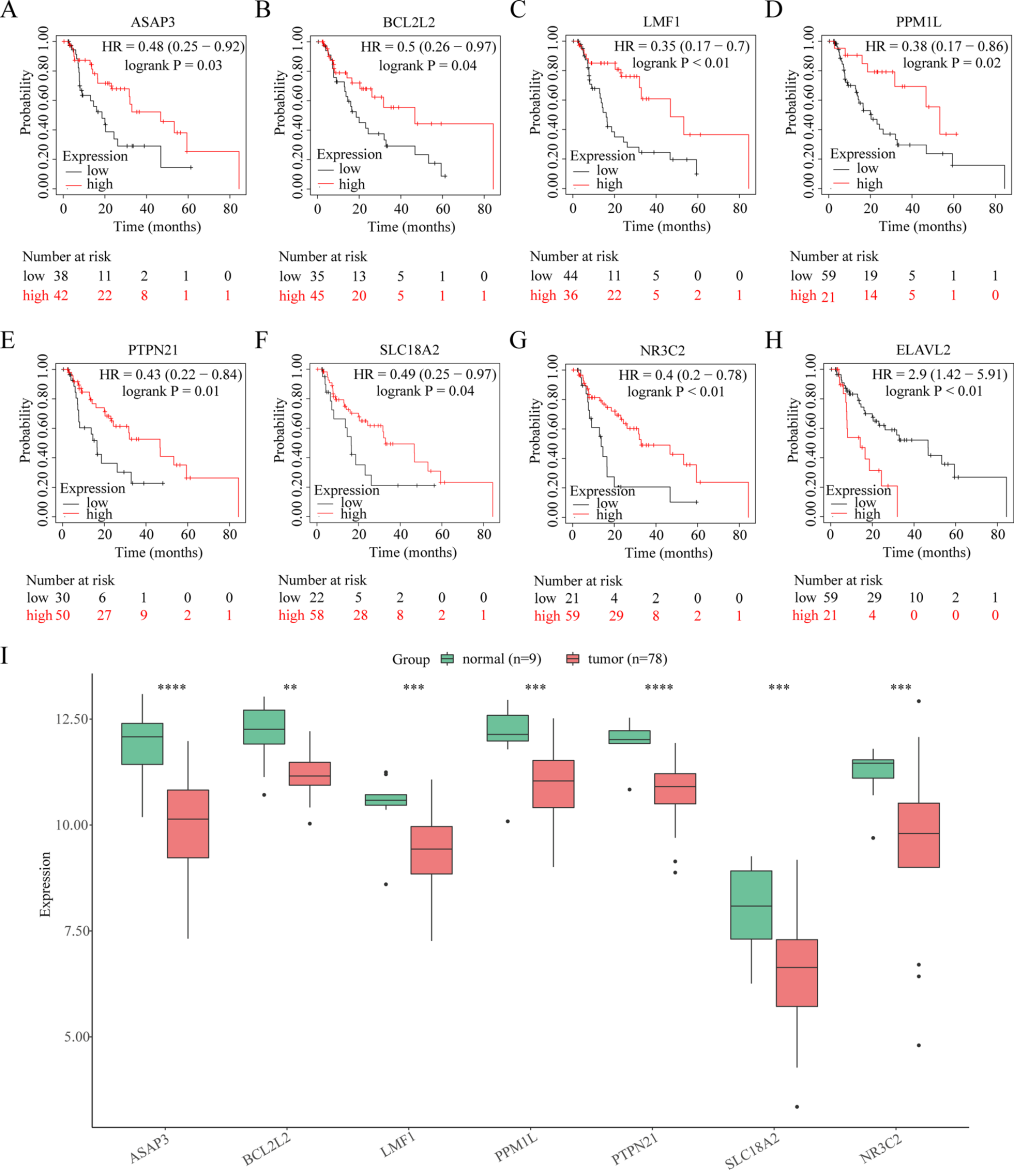
Overall survival of the target genes and their expression in EAC and adjacent tissues. Wilcoxon test *P < 0.05, **P < 0.01, and ***P < 0.001

To explore the prognostic value of downregulated target mRNAs, their OS was assessed using the Kaplan–Meier plotter database. Patients in the low-expression group had a reduced OS compared with those in the high-expression group when the *P-*value was < 0.05 (Fig. 9A–G and Table 2), except for *ELAVL2* (Fig. 9H and Table 2), where the seven target mRNAs in the EAC and adjacent samples and those of they all were poorly expressed (Fig. 9I). Regarding the other target mRNAs, these genes had no prognostic value (*P* < 0.05; Additional file 3: Table 1). Therefore, these data suggested that the signature of the following seven downregulated target mRNAs—*ASAP3, BCL2L2, LMF1, PPM1L, PTPN21, SLC18A2*, and *NR3C2*—have a prognostic value in EAC.

**Table 2:** Prognostic value of the eight mRNAs in patients with esophageal adenocarcinoma

Symbol	HR	95% CI	*P*-value
*ASAP3*	0.48	0.25–0.92	0.03
*BCL2L2*	0.50	0.26–0.97	0.04
*LMF1*	0.35	0.17–0.70	< 0.01
*PPM1L*	0.38	0.17–0.86	0.02
*PTPN21*	0.43	0.22–0.84	0.01
*SLC18A2*	0.49	0.25–0.97	0.04
*ELAVL2*	2.9	1.42–5.91	< 0.01
*NR3C2*	0.4	0.2–0.78	< 0.01

*HR* Hazard ratio, *CI* Confidence interval

## Discussion

In this study, we used bioinformatics, particularly WGCNA, to identify 28 DEMs associated with EAC; two of these miRNAs were then selected to establish the prognostic signature model of EAC via multiple Cox regression analysis.

This study discovered two crucial miRNAs associated with a poor EAC prognosis and constructed a prognostic model comprising these miRNAs that had upregulated expression in the EAC tissues. For the two prognostic miRNAs (mir-550a-1 and mir-421), the mature miRNAs of mir-550a-1, mir-421, miR-421, miR-550a-3p, and miR-550a-5p were identified using the miRbase database. The suppression of miR-421 and miR-550a-3p inhibited the proliferation, invasion, and migration of EAC cells. The unfavorable prognosis of miR-421 in EAC has been reported previously, and upregulated miR-421 expression has been identified as an unfavorable prognostic marker in EAC [26]. The overexpression of miR-421 facilitates cell proliferation in non-small-cell lung cancer (NSCLC) [27]. In addition, the association of miR-421 with various tumors has garnered growing attention. For example, the upregulated miR-421 expression has been shown to inhibit the proliferation and metastasis of colorectal cancer by targeting *MTA1* [28]. Moreover, circAHNAK1 suppresses the proliferation and metastasis of triple-negative breast cancer cells by modulating the miR-421 expression (29). Similarly, circSETD3 acts as a sponge for miR-421 and inhibits the growth of hepatocellular carcinoma [30]. N-Myc has been shown to promote the development of neuroendocrine therapy resistance in prostate cancer through the differential regulation of the miR-421 pathway [31]. Although the prognostic value of miR-421 in EAC and the mechanism of miR-421 in other cancers have been validated, the mechanism through which this operates in EAC remains unclear. The present study shows that the high expression of miR-421 in EAC cells may be a useful prognostic indicator in EAC.

Regarding the two mature miRNAs of mir-550a-1 (miR-550a-3p and miR-550a-5p), to the best of our knowledge, the association of miR-550a-3p and miR-550a-5p with EAC has not been studied previously. miR-550a-3p reportedly shows high expression and is associated with significantly reduced OS in melanoma [32]. The upregulated expression of miR-550a-3p significantly promotes the cellular proliferation, invasion, and migration in NSCLC, whereas the knockdown of the miR-550a-3p expression inhibits cancer growth and metastasis [33]. Recent studies have shown that high expression of miR‐550a‐3p reverses the inhibitory effect of the increased LINC00261 expression, and the reduced *SDPR* expression reverses the growth-promoting effect of miR‐550a‐3p in breast cancer stem cells [34]. Regarding miRNA-550a-5p, a previous study demonstrated that patients with lung adenocarcinoma in the miR-550a-5p high-expression group had a shorter OS than those in the low-expression group [35]. Transfection of an miR-550a-5p mimic promotes cell migration in colorectal cancer [36]. Recent evidence suggests that miR-550a-5p shows high expression and promotes tumor proliferation by binding to *LIMD1* in lung adenocarcinoma [37]. Furthermore, the in vitro expression of miR-550a-3p and miR-550a-5p was higher in EAC cells than in esophageal epithelial cells; however, the expression of miR-550a-3p was more significant, suggesting that miR-550a-3p plays an important role in the prognosis, occurrence, and progression of EAC. Nonetheless, these findings must be validated in future studies.

Metascape database analysis revealed that the target mRNAs *PRKACB* and *PDCD4* of miR-550a-3p as well as *RPS6KA5* and *BCL2L2* of miR-550a-5p were associated with miRNA cancer pathway, providing important clues for subsequent studies on the mechanism underlying EAC development. Unfortunately, no target gene for miR-421 was enriched in miRNA cancer pathway. Nevertheless, these findings should be further explored in subsequent studies. Furthermore, seven prognostic target mRNAs—*ASAP3*, *BCL2L2*, *LMF1*, *PPM1L*, *PTPN21*, *SLC18A2*, and *NR3C2*—were obtained through Kaplan–Meier analysis. Among these mRNAs, the prognostic values and mechanisms of *LMF1*, *PPM1L*, and *PTPN21* have rarely been reported in cancers. To our best knowledge, the correlation between the four other prognostic target mRNAs and EAC has not been studied previously. miRNA-143-3p reportedly inhibits the metastases of colorectal cancer by targeting *ASAP3* [38]. The inhibition of *BCL2L2* results in the high expression of miR-335-5p, which increases cisplatin sensitivity in ovarian cancer [39]. The low expression of *SLC18A2* reduces OS in prostate cancer [40]. Downregulated *NR3C2* expression is associated with a poor prognosis and aggressive characteristics in nondistant metastatic clear-cell renal cell carcinoma [41].

Overall, the consistency of our findings with previous studies confirms the high reliability of the analysis methods used in this study. However, the detailed mechanisms of miR-421, miR-550a-3p, and miR-550a-5p that influence the tumorigenesis and development of EAC by targeting mRNAs require urgent attention.

In conclusion, we analyzed the EAC data obtained from TCGA using combined bioinformatic approaches and cell experiments and identified prognostic indicators of two miRNAs associated with seven prognostic target mRNAs as the possible indicators for future EAC diagnosis and treatment.

## Supplementary Information

**Additional file 1.** Esophageal adenocarcinoma prognostic markers-1.

**Additional file 2.** Esophageal adenocarcinoma prognostic markers-2.

**Additional file 3.** Supplementary Material.

## Data Availability

The original contributions presented in the study are included in the article/Additional files Material, further inquiries can be directed to the corresponding author/s.
