# Supplementary material for: Mir-421 and mir-550a-1 are potential prognostic markers in esophageal adenocarcinoma
Source: Biol Direct. 2023 Feb 24;18:5. doi: 10.1186/s13062-022-00352-8 (PMC9951500; doi:10.1186/s13062-022-00352-8)
Supplement: Supplementary file 3 — Additional file 3. Supplementary Material. [file 13062_2022_352_MOESM3_ESM.docx]

Supplementary Material

**
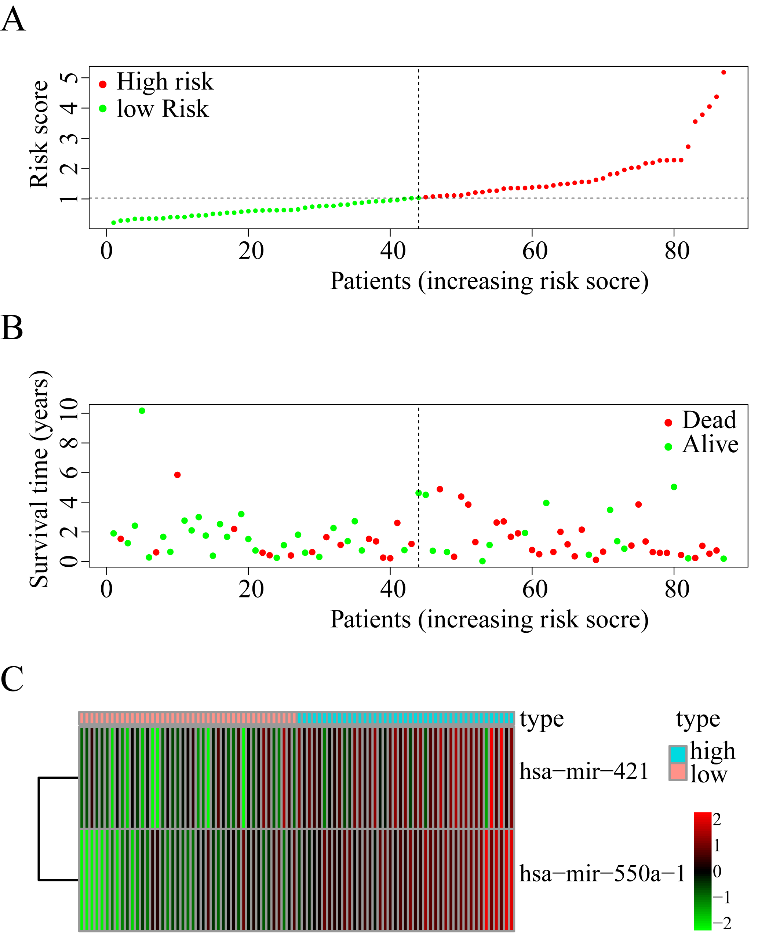
**

**Supplemental Figure 1.** Prognostic model of differentially expressed miRNAs (DEMs) associated with esophageal adenocarcinoma. (A) Distribution of the risk scores. (B) Distribution of patient survival status. (C) Risk score system of the two DEMs in the low- and high-risk groups.


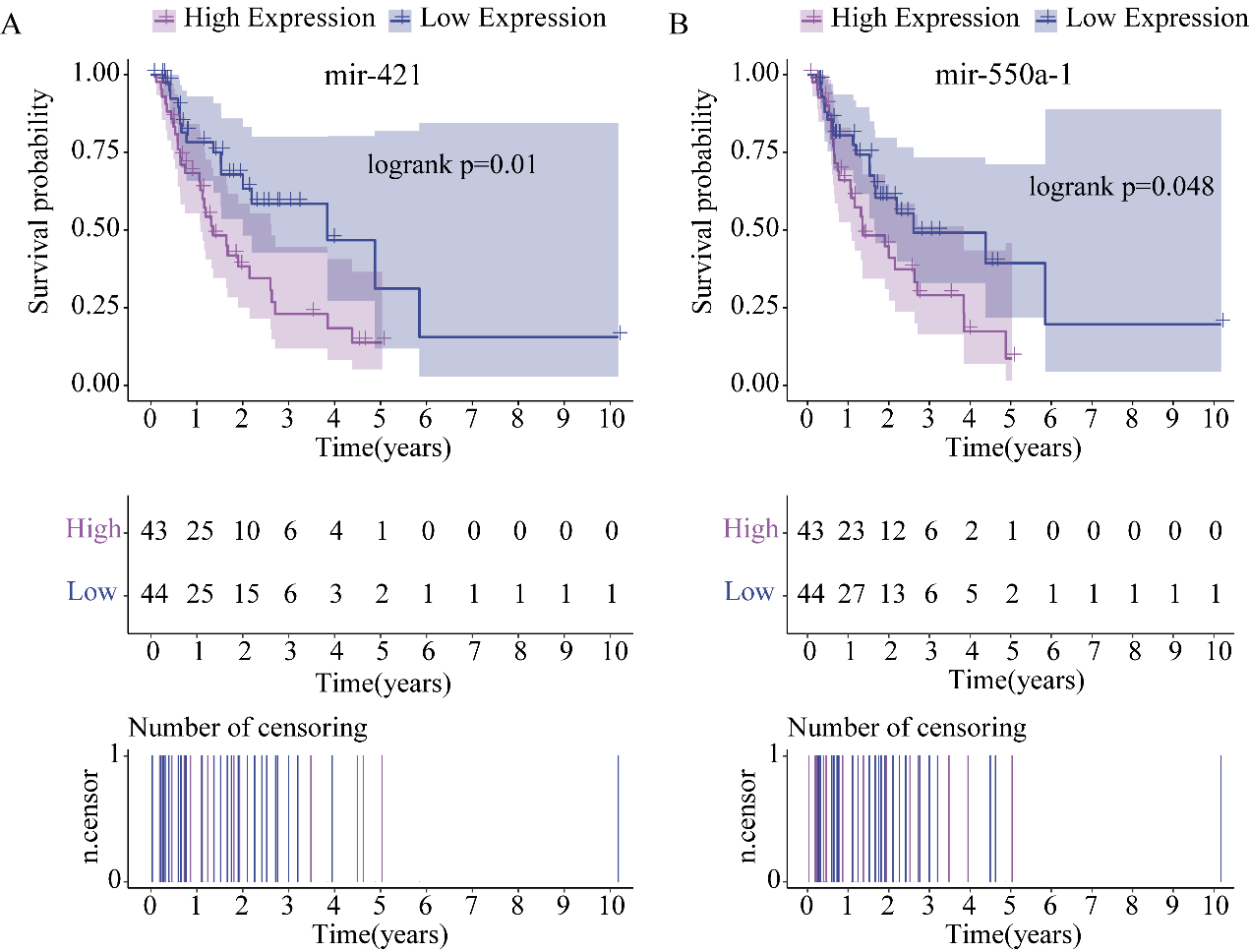


**Supplemental Figure 2.** Overall survival (OS). (A), (B) OS of mir-421 and mir-550a-1, respectively.


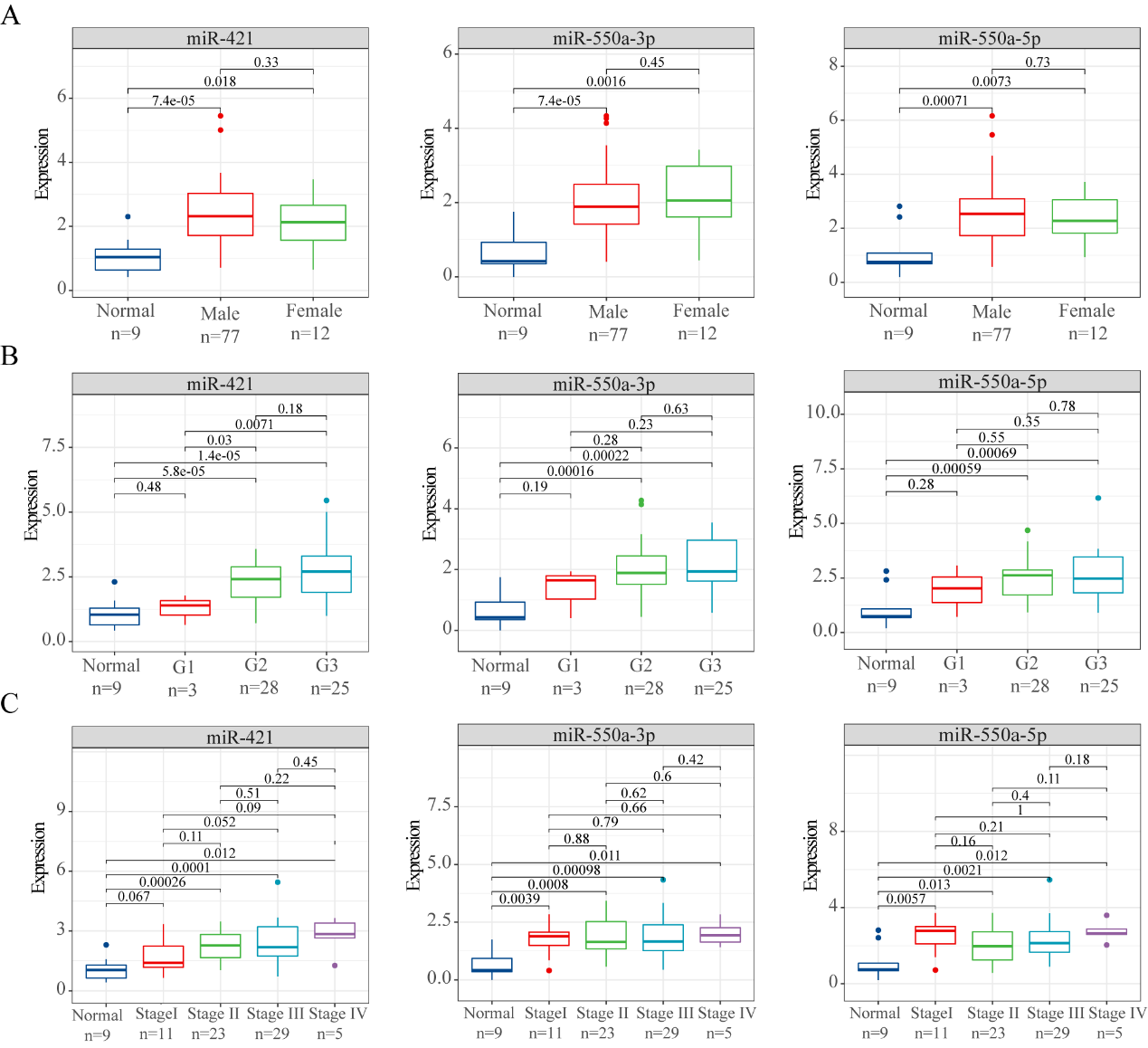


**Supplemental Figure 3.** Expression of the mature miRNAs in EAC subgroup.


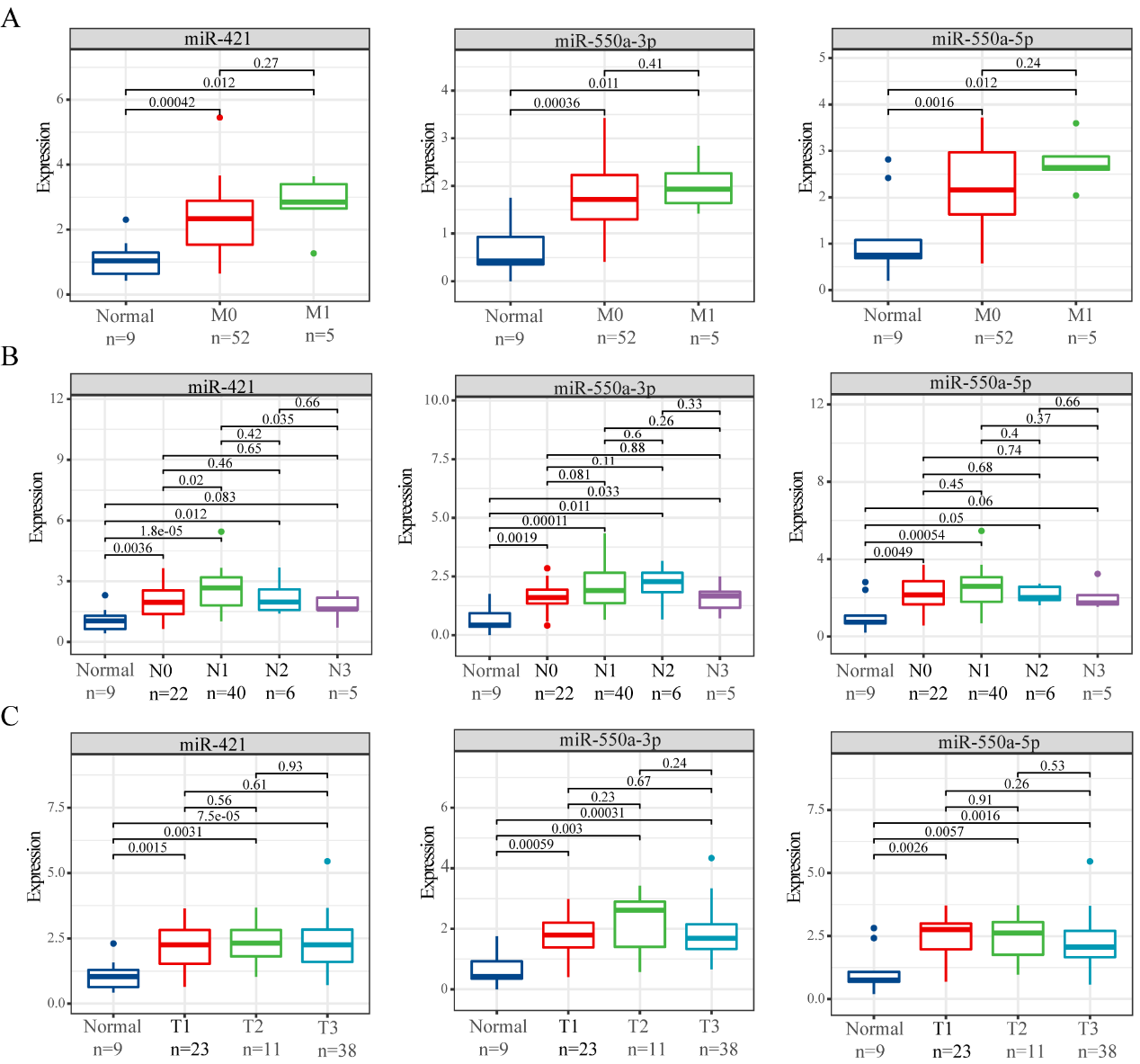


**Supplemental Figure 4.** Expression of the mature miRNAs in EAC subgroup.


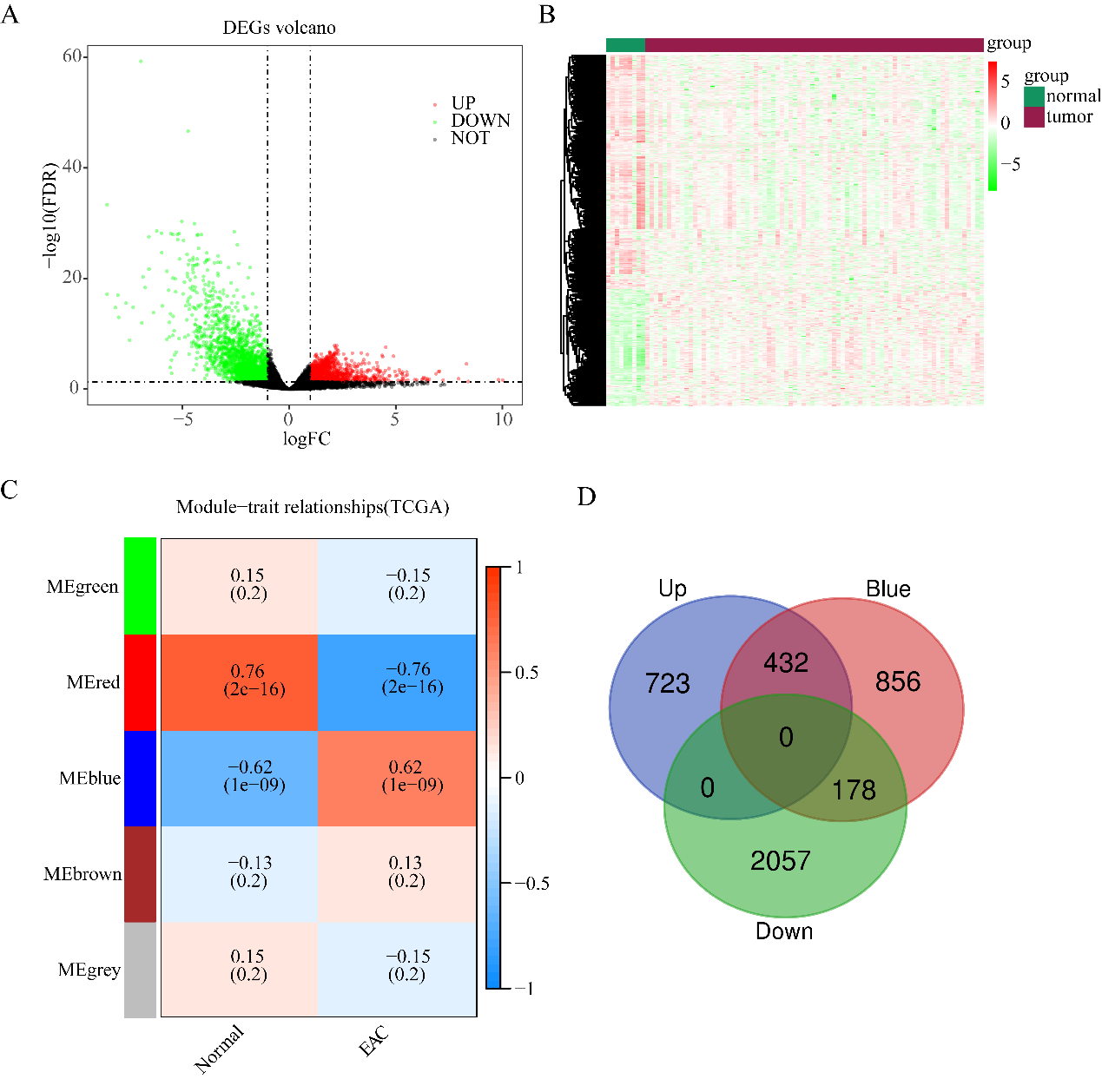


**Supplemental Figure 5.** Identification of differentially expressed genes (DEGs) associated with esophageal adenocarcinoma (EAC). (A) Volcano plots of DEGs. (B) Heatmap of DEGs in both the EAC and adjacent normal samples. (C) Relationship of differentially expressed microRNAs (miRNAs) between the modules of normal samples and those of EAC samples. (D) Venn plots of mRNAs between DEGs and miRNAs in the blue module.

**Supplemental Table 1.** Prognostic value of the 12 mRNAs in patients with esophageal adenocarcinoma.

| Symbol | HR | 95% CI | *P*-value |
| --- | --- | --- | --- |
| *ECHDC2* | 0.71 | 0.36-1.40 | 0.32 |
| *MXL1* | 0.74 | 0.34-1.62 | 0.45 |
| *MYO9A* | 1.38 | 0.61-3.16 | 0.44 |
| *PDCDC4* | 0.66 | 0.33-1.30 | 0.22 |
| *PRKACB* | 0.53 | 0.28-1.02 | 0.05 |
| *REEP1* | 1.46 | 0.73-2.92 | 0.28 |
| *RPS6KA5* | 1.87 | 0.90-3.86 | 0.09 |
| *SCNN1G* | 1.52 | 0.78-2.97 | 0.22 |
| *THRB* | 0.59 | 0.30-1.16 | 0.12 |
| *CPEB3* | 0.73 | 0.36-1.46 | 0.37 |
| *KCNJ15* | 0.74 | 0.39-1.42 | 0.37 |
| *NT5C1B* | 1.58 | 0.83-3.02 | 0.16 |

HR, hazard ratio; CI, confidence interval
